# Immature rats show ovulatory defects similar to those in adult rats lacking prostaglandin and progesterone actions

**DOI:** 10.1186/1477-7827-2-63

**Published:** 2004-09-03

**Authors:** María Gaytan, Carmen Bellido, Concepcion Morales, Marcelino Gonzalez-Padilla, Jose E Sanchez-Criado, Francisco Gaytan

**Affiliations:** 1 Department of Cell Biology, Physiology and Immunology, School of Medicine, University of Cordoba, Spain; 2 Department of Pathology, School of Medicine, University of Cordoba, Spain

## Abstract

Gonadotropin-primed immature rats (GPIR) constitute a widely used model for the study of ovulation. Although the equivalence between the ovulatory process in immature and adult rats is generally assumed, the morphological and functional characteristics of ovulation in immature rats have been scarcely considered. We describe herein the morphological aspects of the ovulatory process in GPIR and their response to classical ovulation inhibitors, such as the inhibitor of prostaglandin (PG) synthesis indomethacin (INDO) and a progesterone (P) receptor (PR) antagonist (RU486). Immature Wistar rats were primed with equine chorionic gonadotropin (eCG) at 21, 23 or 25 days of age, injected with human chorionic gonadotropin (hCG) 48 h later, and sacrificed 16 h after hCG treatment, to assess follicle rupture and ovulation. Surprisingly, GPIR showed age-related ovulatory defects close similar to those in adult rats lacking P and PG actions. Rats primed with eCG at 21 or 23 days of age showed abnormally ruptured corpora lutea in which the cumulus-oocyte complex (COC) was trapped or had been released to the ovarian interstitum, invading the ovarian stroma and blood and lymphatic vessels. Supplementation of immature rats with exogenous P and/or PG of the E series did not significantly inhibit abnormal follicle rupture. Otherwise, ovulatory defects were practically absent in rats primed with eCG at 25 days of age. GPIR treated with INDO showed the same ovulatory alterations than vehicle-treated ones, although affecting to a higher proportion of follicles. Blocking P actions with RU486 increased the number of COC trapped inside corpora lutea and decreased ovulation. The presence of ovulatory defects in GPIR, suggests that the capacity of the immature ovary to undergo the coordinate changes leading to effective ovulation is not fully established in Wistar rats primed with eCG before 25 days of age.

## Introduction

Ovulation, the release of mature oocytes from the ovary, requires proteolytic degradation of the follicle wall, as well as the overlying ovarian tissues. This happens through the expression of a series of critical genes, triggered in a precise temporal and spatial pattern by the preovulatory LH surge [1,2]. It is worthy to note that, for successful ovulation, follicle rupture has to occur just at the site of the follicle wall facing the ovarian surface, thus allowing release of the cumulus-oocyte complex (COC) to the periovarian space, while preventing proteolytic damage of the perifollicular tissues at the basolateral follicle sides. A large amount of information on the ovulatory process was accumulated during the last century (reviewed in [1-5]), and the involvement of crucial genes such as those encoding cyclooxygenase-2 (COX-2), and progesterone receptor (PR) has been clearly established. However, the mechanisms underlying the spatial targeting of the follicle rupture remain poorly understood. Although mechanical factors are likely involved in stigma formation and rupture [6], the mechanisms responsible for the specific location of proteolytic breakdown of the theca layers and perifollicular connective tissue at the apex of the follicle are not known. In recent studies [7-9] we have proposed that both prostaglandins (PG) and progesterone (P), classically recognized as essential ovulatory factors [1,2], play complementary roles in the spatial targeting of follicle rupture. This was supported by detailed morphological studies in cycling rats treated with indomethacin (INDO), a strong inhibitor of PG synthesis, and RU486 (a PR antagonist), showing antiovulatory effects [1,2,10-12].

Gonadotropin-primed immature rats (GPIR) constitute a useful model for the study of ovulation. The administration of a single dose of equine chorionic gonadotropin (eCG) to immature animals induces the growth of abundant follicles, that reach preovulatory size in two days. Ovulation is then triggered by a single dose of human chorionic gonadotropin (hCG), thus providing a large number of synchronized ovulatory follicles [13-25]. An additional advantage of this model is the absence of regressing corpora lutea of previous cycles. This is relevant because structural luteolysis, that is temporally coincident with ovulation in cycling rats, also involves tissue remodeling and proteolytic degradation of the extracellular matrix [5]. For these reasons, GPIR (ranging from 21 to 28 days of age, at the time of eCG treatment [13-25]), have been widely used in studies focused on the ovulatory process, and a large amount of the information in this topic is derived from studies in immature rats. However, it should be kept in mind that GPIR constitute a non-physiological model and the possible immaturity of the pathways leading to ovulation cannot be ruled out.

In order to examine further the role of PG and P in follicle rupture and ovulation, we performed detailed morphological analysis of the ovulatory process in inmature rats primed with gonadotropins at different ages. Surprisingly, these animals showed age-related alterations of the follicle rupture similar to those of adult rats lacking PG and P actions. We report herein the morphological alterations of follicle rupture and ovulation in GPIR and the effects of treatment with P and/or PG of the E series, as well as the response of GPIR to PG synthesis inhibition with INDO and to a PR antagonist (RU486), whose inhibitory effects in ovulation are clearly established [1,2,8,13,14].

## Materials and methods

### Animals and drugs

Wistar female rats bred in the vivarium of the University of Cordoba were used. The day the litters were born was considered as day 0. Litter size was adjusted to 8 pups. The animals were maintained under controlled conditions of light (14L:10D; lights on 0500-1900) and temperature (22°C). The animals had free access to pelleted food and tap water. Experimental procedures were approved by the Cordoba University Ethical Committee for animal experimentation and were conducted in accordance with the European Union guidelines for care and use of experimental animals. Indomethacin (INDO), Progesterone (P), equine chorionic gonadotropin (eCG), human chorionic gonadotropin (hCG) and prostaglandins were purchased from Sigma.(St Louis, MO). The progesterone antagonist RU486 was obtained from Exlegin (Paris, France).

### Gonadotropin-priming at different ages

Wistar immature rats were injected sc with 10 IU of eCG at 1700 h at 21, 23 or 25 days of age, and 48 h later were injected sc with 10 IU of hCG, a frequently used schedule [23,26,27]. Body weights were in the recommended range [23,26] (46.0 ± 0.34, 57.0 ± 1.30 and 61.8 ± 1.4 g, mean ± SEM for n = 5). The animals (5 per group) were sacrificed at 0900 h on the day following hCG injection (i.e. at 24, 26 and 28 days of age). The ovaries, including the ovarian bursa, oviducts and periovarian fat pad, were fixed in Bouin-Hollande's fluid for 24 h and processed for paraffin embedding.

### Gonadotropin-primed rats treated with prostaglandins and/or progesterone

Immature rats were primed with eCG at 21 days of age and with hCG 48 h later as described above. In addition, these animals were injected with 100 μg of PGE1 or PGE2, 1 mg of P or 1 mg of P plus 100 μg of PG E1 or PGE2 or vehicles (70% ethanol in saline) at 1730 h (30 min after hCG treatment). These dosages were effective, in the same body weight basis, in previous studies in adult rats [9]. The animals (5 per group) were killed at 0900 h on the day after hCG treatment (i.e. at 24 days of age) and the ovaries were processed as described above.

### Gonadotropin.primed rats treated with indomethacin or RU486

Immature rats were primed with eCG at 23 days of age and with hCG 48 h later as described in previous experiments. Indomethacin-treated rats received a sc injection of 0.5 mg of INDO or vehicle (olive oil) at 1200 h at 25 days of age. RU486-treated rats were injected sc with 0.5 mg of RU486 or vehicle (olive oil) at 0900 h at 24 and 25 days of age.The animals (5 per group) were killed at 0900 h on the day following hCG injection (i.e. at 26 days of age) and the ovaries were processed as described above.

### Histological analysis of follicle rupture and ovulation

The right ovaries were serially sectioned (6 μm) and stained with hematoxylin and eosin. All sections were examined under the microscope. The total number of cumulus-oocyte complexes (COCs) per ovary were counted. The number of COCs trapped inside the corpus luteum, released to the ovarian interstitium, retained in the bursal cavity or found in the oviducts, was recorded. COCs released to the ovarian interstitium were clearly recognizable by the presence of the oocyte in metaphase II and the first polar body, dispersed cumulus and follicular fluid. The total number of COCs per ovary matches the total number of corpora lutea (unruptured or not). In addition to absolute values, the number of COCs in each location was expressed as the percentage with respect to the total number of COCs (or corpora lutea) per ovary, to assess ovulatory efficiency, avoiding variability in the total numbers of corpora lutea among the different groups. Unruptured follicles measuring less than 575 μm in diameter, showing signs of atresia, such as irregularities of the granulosa cell layer, presence of apoptotic granulosa cells, as well as lack of dispersion of the cumulus or resumption of meiosis, were not considered.

Statistical analysis was performed by ANOVA followed by the Student-Newman-Keuls method for multiple comparison among means. Significance was considered at the 0.05 level.

## Results

### Ovulatory process in immature rats primed at different ages

**Figure 1:**
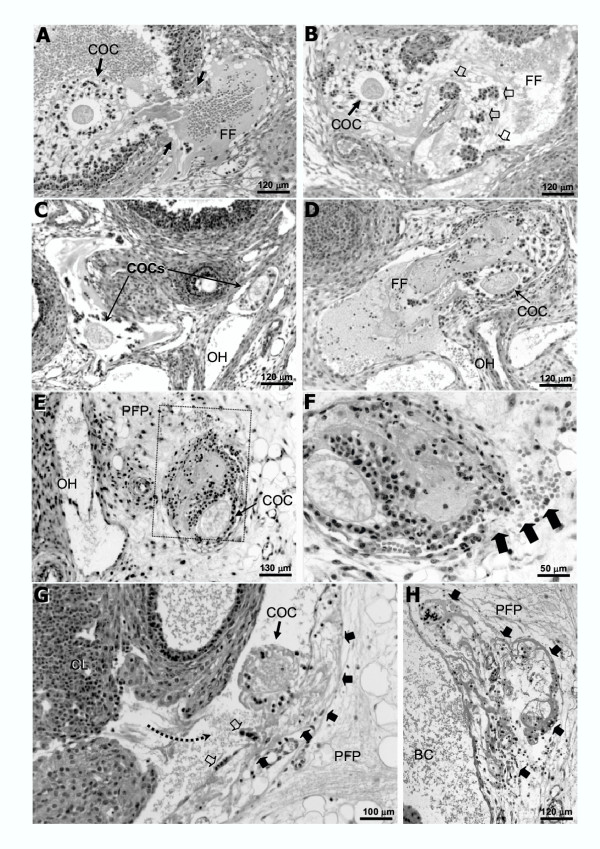
Representative micrographs from the ovary of immature rats primed with eCG at 21 (*A-D*, *G, H*) or 23 (*E, F*) days of age, stained with hematoxylin and eosin. **A**, luteinized follicle showing trapped COC and release of follicular fluid (*FF*) to the ovarian interstitium. The rupture of the theca layers are indicated by *arrows*. **B**, COC released to the ovarian interstitium, in a lacunae of follicular fluid (*FF*). Clusters of granulosa cells are indicated by *open arrows*. **C, D**, COCs in the lymphatic (*A*) or blood (*D*) vessels at the ovarian hilus (*OH*). **E, F**, COC inside a blood vessel located in the periovarian fat pad (*PFP*) near the ovarian hilus (*OH*). The framed area is shown at higher magnification in *F *showing rupture of the blood vessel and escape of red blood cells (*arrows*). **G, H**, Non-consecutive serial sections showing a COC released to the bursal cavity (*BC*), adhered to the ovarian bursa. Degradation of the ovarian bursa by follicular fluid and granulosa cells (*open arrows*) and invasion (*arrows*) of the periovarian fat pad (*PFP*) can be observed.

**Figure 2:**
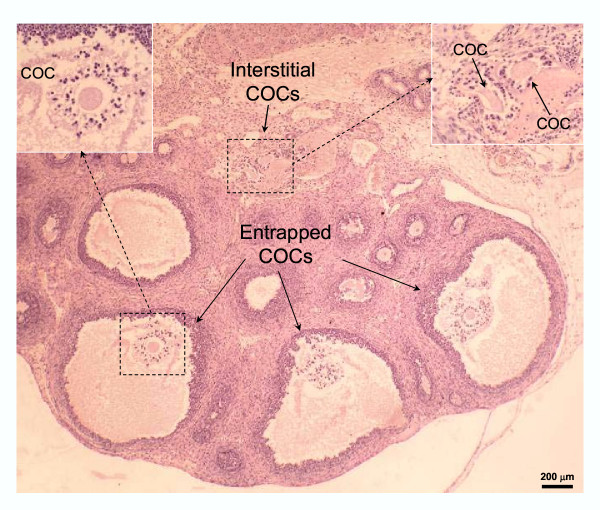
Micrograph of the ovary of a rat primed with eCG at 21 days of age, stained with hematoxylin and eosin. Several COCs trapped inside corpora lutea, showing dispersion of the cumulus, and interstitial COCs inside a blood vessel.

**Figure 3:**
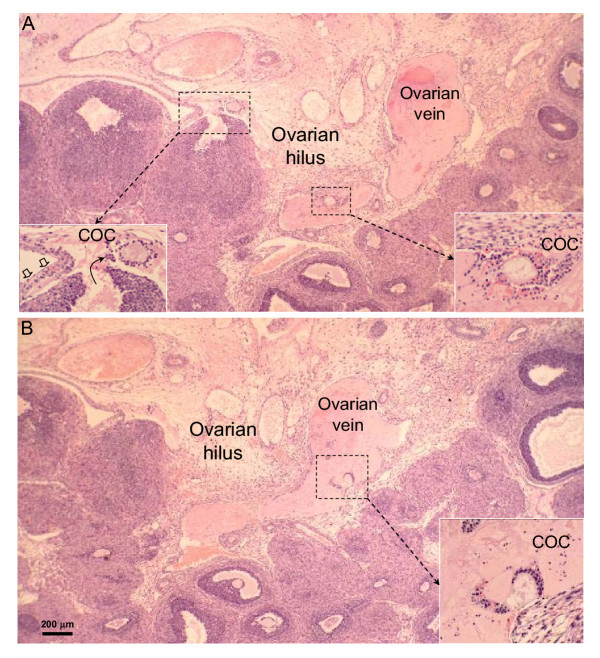
Non-consecutive serial sections of the ovary of a rat primed with eCG at 21 days of age and stained with hematoxylin and eosin. The ovarian vein is embolized with folicular fluid and two COCs (in **A **and **B**). A COC released to the ovarian hilus can be also observed in **A**. The ovarian surface is indicated by *empty arrows *in the *inset*.

**Figure 4:**
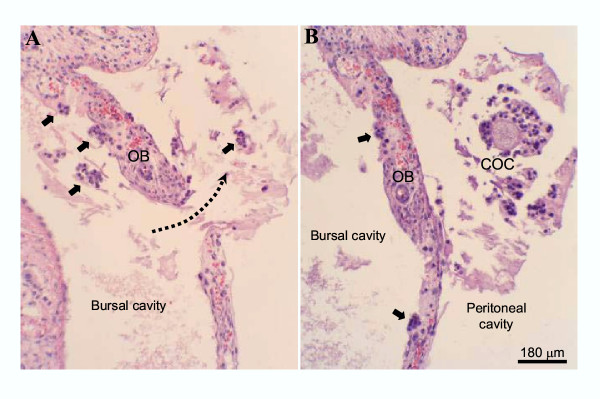
Micrographs from non-consecutive serial sections from the ovary of an immature rat primed with eCG at 21 days of age. The ovarian bursa (*OB*) has been degraded (**A**) allowing release of the COC to the peritoneal cavity (**B**) Clusters of granulosa cells (*arrows*) can be observed free or attached to the ovarian bursa. Hematoxylin and eosin.

**Figure 5:**
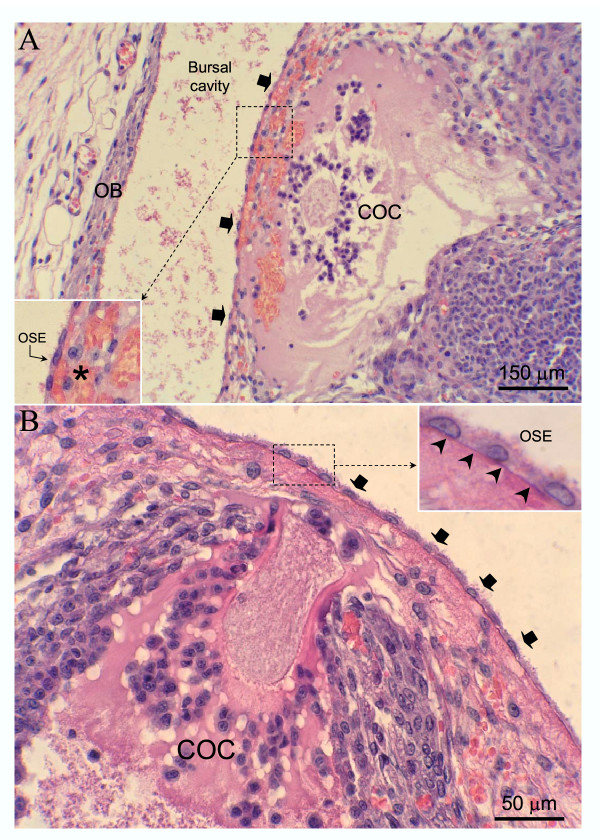
Representative micrographs from the ovary of immature rats primed with eCG at 21 (*A*) or 23 (*B*) days of age, showing luteinized follicles ruptured at the apex. The ovarian surface epithelium (*OSE, arrows in A and B*), and its basement membrane (*arrowheads in B*) remain intact, whereas the underlying ovarian tissue has been degraded (*asterisk in A*). The COC is retained under the ovarian surface. *OB*, ovarian bursa. Hematoxylin and eosin.

**Figure 6:**
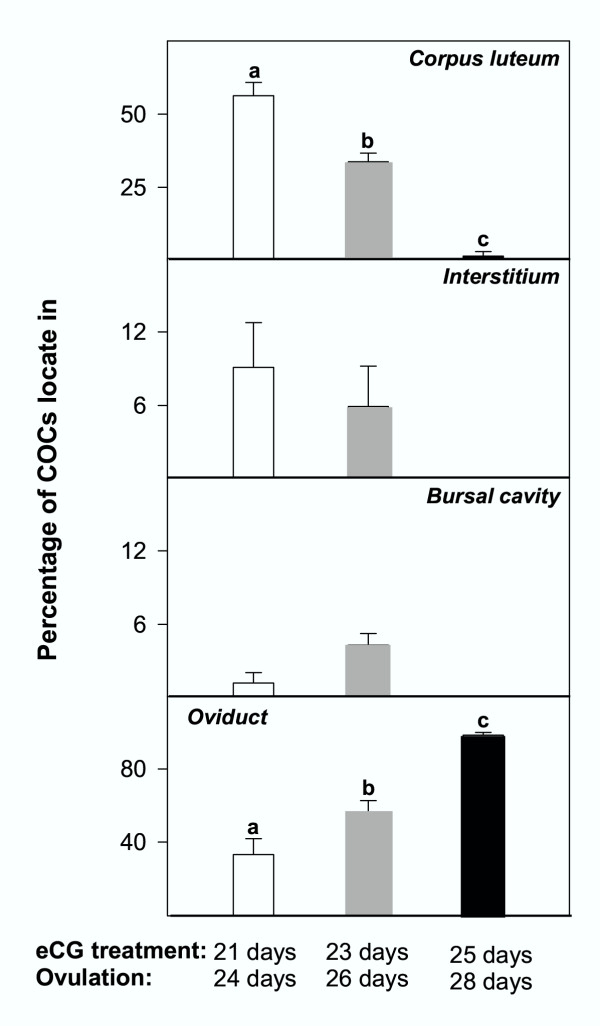
Percentage of cumulus-oocyte complexes (COCs) trapped inside the corpus luteum, released to the interstitium, located in the bursal cavity, or in the oviducts, in immature rats primed with eCG at 21, 23 or 25 days of age and ovulating at 24, 26 or 28 days of age respectively. See Table 1 for absolute counts. Different superscripts mean significant (p < 0.05) differences.

Absolute numbers of COCs, that match the numbers of corpora lutea per ovary, are presented in Table 1, whereas relative data (the proportion of COCs found in each specific location) are shown in Fig. 6. On the day following hCG treatment, the total number of COCs per ovary was equivalent for the different age-groups (Table 1). Immature rats primed with eCG at 21 or 23 days of age showed frequent ovulatory defects (see Figs 1,2,3,4,5 for representative micrographs and Fig. 6. for quantitative data). The COC was trapped in about 60% and 40% of the luteinized follicles in rats primed with eCG at 21 and 23 days of age respectively (Figs. 1A, 2, 6). However, many luteinized follicles containing the COC showed rupture of the theca layers and release of follicular fluid to the ovarian interstitium (Fig. 1A). Furthermore, 6–10% of the COCs were released to the ovarian interstitum (Figs. 1B, 2, 3, 6). In these cases, degradation of the ovarian stroma with invasion of the blood and lymphatic vessels and formation of emboli containing the COC and follicular fluid (Figs 1C,1D,1E,1F, 2, 3) were observed. In some animals, massive embolism of the ovarian vein at the ovarian hilus with follicular fluid and COCs (Fig. 3) caused ovarian hyperemia and vascular congestion. In addition, proteolytic degradation of the ovarian bursa by follicular fluid and granulosa cells, with invasion of the periovarian fat pad (Fig. 1G,1H) and even release of the COC to the peritoneal cavity (Fig. 4), was also observed. A characteristic feature of some corpora lutea ruptured at the apex, was the presence of an unruptured ovarian surface epithelium, and the COC was trapped in lacunae of follicular fluid and blood in the tunica albuginea (Fig. 5). Variable numbers of COCs (from 0 to 25%) were located in the bursal cavity (Fig. 6). From 33% to 60% of COCs were found in the oviducts, in rats primed with eCG at 21 and 23 days of age respectively. Otherwise, in immature rats primed with eCG at 25 days of age, the vast majority of COCs were found in the oviducts and abnormally ruptured corpora lutea (with trapped COC) were only occasionally found.

**Table 1:** Number of COCs per ovary in control rats primed with eCG at different ages.

**Age (days) at eCG treatment**	**Total COCs**	**COCs located in**			
		
		Corpus luteum	Interstitium	Ovarian Bursa	Oviduct
21	42.5 ± 8.72	22.5 ± 4.32	3.17 ± 1.47	0.67 ± 0.42	16.2 ± 6.93
23	58.4 ± 4.74	18.8 ± 0.70	3.20 ± 1.74	2.40 ± 0.51	34.0 ± 6.54
25	38.6 ± 3.76	0.6 ± 0.60^a,b^	--	--	37.4 ± 3.20

Values are the mean ± SEM for n = 5. ANOVA and Student-Newman-Keuls multiple range test. ^a ^p < 0.05 *vs *21 days. ^b ^p < 0.05 *vs *23 days

### Effects of P and/or PGE treatment

**Figure 7:**
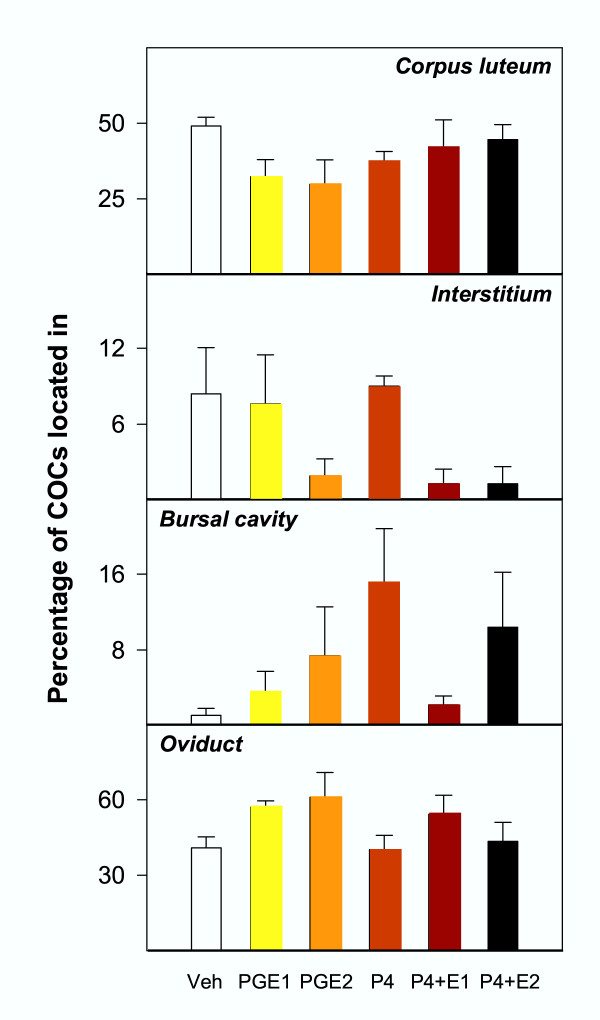
Percentage of cumulus-oocyte complexes (COCs) trapped inside the corpus luteum, released to the interstitium, located in the bursal cavity, or in the oviducts, in rats primed with e CG at 21 days of age and injected two days later with vehicles (Veh), prostaglandin E1 (PGE1), prostaglandin E2 (PGE2), progesterone (P) or progesterone plus PGE1 or PGE2. See Table 2 for absolute counts. No significant differences were found.

The total number of COCs per ovary was equivalent in rats primed with eCG at 21 days of age and treated with vehicles, P, PGE1, PGE2, P plus PGE1 or P plus PGE2 (Table 2). Neither P, PG or combined P plus PG treatment, prevented ovulatory defects and these animals showed the same morphological alterations as gonadotropin and vehicle-treated rats. However, degradation of the interstitial tissue and embolism of blood vessels with follicular fluid and granulosa cells were very scarce in prostaglandin-treated animals. Although the number of effectively ovulated oocytes were apparently increased in prostaglandin-treated rats, differences were not large enough to be statistically significant (Fig. 7).

**Table 2:** Number of COCs per ovary in rats primed with eCG at 21 days of ages.

**Treatment**	**Total COCs**	**COCs located in**			
		
		Corpus luteum	Interstitium	Ovarian Bursa	Oviduct
Vehicles	35.4 ± 3.52	17.2 ± 1.47	2.6 ± 0.85	0.6 ± 0.24	15.0 ± 2.72
PGE_1_	33.2 ± 4.20	11.0 ± 2.42	2.3 ± 1.02	1.2 ± 0.58	19.0 ± 3.40
PGE_2_	33.2 ± 1.62	10.2 ± 2.80	0.6 ± 0.39	3.6 ± 2.59	18.8 ± 3.38
P_4_	45.6 ± 7.77	17.2 ± 2.97	3.4 ± 1.06	5.8 ± 3.10	19.2 ± 5.45
P_4 _+ PGE_1_	34.6 ± 5.17	14.6 ± 3.37	0.6 ± 0.39	0.8 ± 0.37	18.6 ± 2.82
P_4 _+ PGE_2_	35.2 ± 6.85	15.6 ± 3.10	0.6 ± 0.39	5.7 ± 2.85	14.2 ± 1.63

Values are the mean ± SEM for n= 5.

### Effects of INDO or RU486 treatments

**Figure 8:**
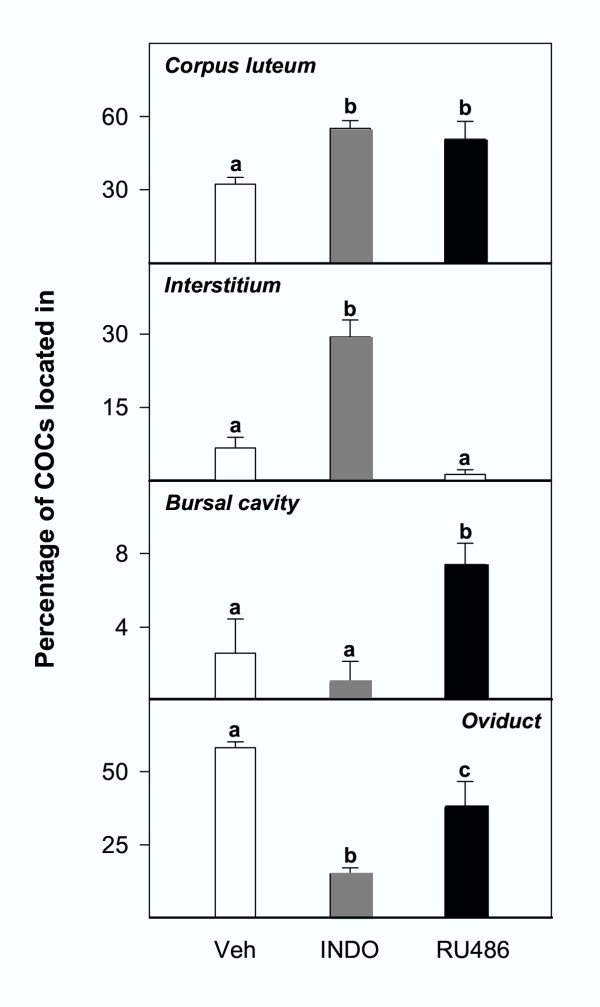
Percentage of cumulus-oocyte complexes (COCs) trapped inside the corpus luteum, released to the interstitium, located in the bursal cavity, or in the oviducts, in rats primed with eCG at 23 days of age and treated with indomethacin (INDO) or RU486. see Table 3 for absolute counts. Different superscripts mean significant (p < 0.05) differences.

The total number of COCs per ovary was equivalent in rats treated with vehicle, INDO or RU486 (Table 3). Immature rats treated with INDO during the preovulatory period showed the same morphological alterations of the ovulatory process as gonadotropin and vehicle-treated rats, although a higher proportion of follicles were affected (Fig. 8). A significantly (p < 0.05) higher number of COCs were released to the ovarian interstitium, whereas the number of COCs found in the oviducts was significantly (p < 0.05) decreased. Rats treated with RU486 showed increased numbers of COCs retained inside the follicle (Fig. 8), whereas the number of COCs released to the interstitium or effectively ovulated were significantly decreased. Most of the luteinized follicles in which the COC was trapped, did not show evident rupture of the theca layers. The numbers of COC trapped in the bursal cavity was significantly increased (Table 3 and Fig. 8).

**Table 3:** Number of COCs per ovary in rats primed with eCG at 23 days of ages.

**Treatment**	**Total COCs**	**COCs located in**			
		
		Corpus luteum	Interstitium	Ovarian Bursa	Oviduct
Vehicle	39.6 ± 1.60	12.8 ± 1.0	2.6 ± 1.73	1.0 ± 0.62	23.2 ± 1.53
INDO	34.4 ± 3.76	19.2 ± 2.55	10.0 ± 1.35^a^	0.4 ± 0.39	5.0 ± 0.05^a^
RU486	41.8 ± 3.86	22.8 ± 4.80	0.6 ± 0.39^b^	3.2 ± 0.57^a,b^	15.2 ± 3.10^a,b^

Values are the mean ± SEM for n = 5. ANOVA and Student-Newman-Keuls multiple range test. ^a ^p < 0.05 *vs *vehicle, ^b ^p < 0.05 *vs *INDO.

## Discussion

Gonadotropin-primed immature rats (GPIR) is a popular model for the study of ovulation. In this model, eCG stimulates the growth of a large cohort of follicles, that reach preovulatory size in about 48 h and are then induced to ovulate by hCG [1,2,28]. Early data indicate that the ovulation rate in immature rats was age-dependent, and that the number of ova found in the oviducts increases, although not linearly, with age [29,30]. However, these early studies do not provide information on the number of ova that were not effectively ovulated, and the marked variability in the rate of ovulation at different ages could be due to differences in the number of recruited growing follicles, in the responsiveness of preovulatory follicles to the ovulatory stimulus or both. In the present study, we evaluated the ovulatory process in immature rats primed with gonadotropins during the 21–25 day-old period, commonly used in studies focused on ovulation [13,17,18,20-23,25,26]. In agreement with previous data [29,30], the proportion of COCs found in the oviducts (i.e. effectively ovulated) increases in parallel to age. Detailed morphological evaluation indicated that ovulation was unsuccessful in 30–60% of preovulatory follicles in rats primed with eCG before 25 days of age. The nearly normal ovulatory process in immature rats primed with eCG at 25 days of age indicates that ovulatory defects were not due to gonadotropin treatment itself, but to ovarian immaturity at earlier ages. The ovulation failure described herein (in rats primed with eCG at 21 or 23 days of age) was not due to decreased recruitment of growing follicles, as indicated by the equivalent numbers of follicles that reach preovulatory size at all ages tested. The data of this study strongly suggest that the relative ovulatory incompetence of young immature rats was due to a defective capacity of preovulatory follicles to undergo the coordinate network of interactions that leads, in reponse to hCG, to COC release to the periovarian space.

Defective ovulation in GPIR was related to abnormal follicle rupture. Whereas some LH-driven morphological changes in preovulatory follicles, such as cumulus expansion, resumption of the meiotic process and initial luteinization were present and showed normal features in GPIR, aberrant follicle ruptures were frequently observed. This was indicated by the presence of COCs released to the ovarian interstitium, and of corpora lutea showing rupture of the theca layers at any site of the follicle wall with release of follicular fluid to the ovarian stroma. The presence of follicle ruptures at the basolateral follicle sides suggests that the mechanisms underlying the spatial targeting of follicle rupture at the apex are not fully established in immature rats primed with eCG before 25 days of age. Almost identical ovulatory defects have been previously reported in INDO-treated adult cycling rats that also show abnormally ruptured follicles and proteolytic degradation of perifollicular tissues [7-9]. Furthermore, INDO-treated GPIR in the present study showed similar morphological alterations of the ovulatory process as vehicle-treated GPIR, even though the drug increased the number of affected follicles. The similarity of the ovulatory alterations found in GPIR and INDO-treated adult rats raises the question of whether the COX-2-prostaglandin pathway is fully established or not in GPIR. In this context, treatment of GPIR with PG of the E series was carried out in order to analyse whether ovulatory defects in GPIR were due to defective PG synthesis. Treatment with prostaglandin E resulted in a decrease in the invasion of the ovarian stroma and blood vessels by follicular fluid and granulosa cells, but ovulation was not improved significantly. In contrast, supplementation with exogenous PG of the E series inhibits abnormal follilce rupture and restores ovulation in INDO-treated adult rats [2,8,31]. This suggests that the possible immaturity of the COX-2-prostaglandin pathway in GPIR would be beyond prostaglandin synthesis, in agreement with previous studies reporting normal PG generation in GPIR in response to hCG treatment [28]. Otherwise, recent studies in COX-2 defective mice [32] indicate that the COX-2-PG pathway is not fully established in immature rodents.

It has been clearly established that activation of PR plays a crucial role in ovulation [10,11,33-37]. Accordingly, adult cycling rats treated with PR antagonists [11] or inhibitors of the P synthesis [34] during the preovulatory period showed almost complete inhibition of follicular rupture [9,11,34]. Noteworthy, the response of GPIR to PR blockage (showing incomplete inhibition of follicle rupture) was similar to that of adult rats lacking both P and PG actions. In this sense, combined treatment of adult rats with RU486 and INDO resulted in follicle rupture in about 25% of preovulatory follicles [9]. Furthermore, some morphological features of the ovulatory process in GPIR, such as the persistence of the ovarian surface epithelium in spite of the degradation of the underlying tissues, was also a characteristic feature of adult rats treated with both INDO and RU486 [9]. However, exogenous P supplementation did not restore ovulation in GPIR, that suggest a defective response of preovulatory follicles to the ovulatory LH-dependent P surge. RU486-treated GPIR showed a significant increase in the number of COCs retained into the bursal cavity. Alterations in the transport of COCs to the oviduct has been previously described in adult rats treated with a PR antagonist [11], although the existence of a role for P in the COC transport to the oviduct cannot be ascertained from the present data.

The end-point of the complex interaction network leading to follicle rupture and COC release, is the expression/activation of proteolytic enzymes, reponsible for extracellular matrix breakdown [24,38,39]. Proteolytic activity has to be closely modulated by protease inhibitors to prevent damage to perifollicular tissues. However, the characterization and regulation of ovarian proteolytic inhibitors has not been completely elucidated. The presence in GPIR of degradation of the ovarian stroma, invasion of blood and lymphatic vessels in abnormally ruptured follicles, strongly suggest that defective ovulation in GPIR was not due to decreased proteolytic activity but due to untargeted proteolysis. This was particularly suggested by the degradation of the ovarian bursa and invasion of the periovarian fat pad by cumulus cells and follicular fluid released at the follicle apex (see Figs. 1G,1H, 3 and 4), permitting, in some cases, escape of the COC to the peritoneal cavity. In this sense, the use of very young immature rats to characterize proteolytic activity regulation during ovulation could provide conflicting data. In this study, we used Wistar rats. The possible existence of slight differences in the age of ovarian maturation among different rat strains cannot be ruled out.

In conclusion, the presence of aberrant ovulations in very young GPIR, closely resembling those in adult rats lacking P and PG actions, strongly suggests that the capacity of the immature ovary to undergo changes leading to effective ovulation is not fully established in Wistar rats primed with eCG before 25 days of age. This should be taken into account when using the GPIR model for studies focused on ovulation.
